# Causes of medical disputes in China and Japan from physicians' perspectives: a qualitative study

**DOI:** 10.3389/frhs.2026.1826004

**Published:** 2026-08-27

**Authors:** Hua Xu, Atsushi Asai, Masao Tabata, Yasuhiro Kadooka

**Affiliations:** 1 Tohoku Daigaku Daigakuin Igakukei Kenkyuka Igakubu, Sendai, Japan; 2 Tohoku University Hospital, Sendai, Japan; 3 Kumamoto University Graduate School of Medicine, Kumamoto, Japan

**Keywords:** causal network of medical disputes, causes for medical disputes, China, cross-cultural study, distrust, doctor-patient relationships, Japan

## Abstract

**Background:**

Medical disputes and strained doctor–patient relationships (DPRs) remain pressing challenges in China. This comparative qualitative study examined Chinese and Japanese doctors' perspectives on the causes of medical disputes to identify context-specific factors, elucidate causal mechanisms, and inform strategies for improvement.

**Method:**

Using purposive sampling, 20 practicing physicians from each country were recruited primarily through the authors' personal networks. Semi-structured interviews were conducted, and qualitative descriptive analysis was used to identify categories and subcategories of perceived contributors to medical disputes.

**Result:**

Five categories and seventeen subcategories emerged. While both countries' doctors identified similar negative factors affecting DPRs, their manifestations differed, explaining divergence in dispute prevalence. In China, disputes were additionally influenced by inefficiencies in medical insurance and the medical dispute resolution system, poverty, healthcare consumerism, distrust, low social morality, and a “vicious cycle of disputes.” In Japan, disputes largely reflected poor communication and mutual distrust rather than broader systemic issues.

**Discussion:**

The findings underscore that medical disputes arise from broader social, medical, legal, economic, cultural, and ethical structures. In China, resolving disputes and improving DPRs requires systemic reforms, including strengthened social security, refined healthcare and legal systems, and enhanced ethics and public education. Sustainable trust-building is thus a long-term, structural endeavor rather than a matter of isolated interactions.

## 1 Introduction

Medical disputes refer to conflicts or disagreements between patients (or their families) and healthcare providers or medical institutions arising from medical processes, clinical practices, treatment outcomes, or inadequate communication (1, 2). In contemporary practice, both China and Japan have experienced increasing tensions in doctor–patient relationships (DPRs), a rising number of disputes associated with medical accidents, and growing incidents of violence against healthcare professionals (3–6). This issue is particularly severe in China. The level of tension in DPRs in China is markedly higher than that in Japan, with medical disputes predominantly concentrated in large medical institutions such as university hospitals (7–9). By contrast, DPRs in Japan are relatively stable overall, with a lower incidence of medical disputes that are more evenly distributed across different types of healthcare institutions (10, 11). These differences suggest that medical disputes should be understood not only at the interpersonal level but also within broader institutional, sociocultural, and systemic contexts.

Existing research has shown that medical disputes arise from the interaction of multiple stakeholders and structural factors. From physicians' perspectives, contributing factors include ineffective communication, inadequate information disclosure and shared decision-making, poor professional attitudes, insufficient clinical competence, medical errors, and inappropriate responses to adverse events such as concealment or dishonesty (11–13). From patients' perspectives, disputes may stem from misunderstandings, unrealistic expectations of treatment outcomes, limited medical knowledge, poor understanding of medical uncertainty, and difficulty distinguishing complications from malpractice (14). In addition, distrust in healthcare institutions, the consumerization of healthcare, media misreporting of medical disputes, and high medical costs may further intensify tensions (15, 16).

Nevertheless, several limitations remain. First, previous studies often treat medical disputes as isolated events rather than dynamic processes, with insufficient attention to their developmental and cumulative nature. Drawing on systems theory and path-dependence theory, medical disputes can be better understood as evolving processes shaped by sequential interactions across stages of care (17, 18). Early communication failures, unmet expectations, or institutional responses may progressively reduce opportunities for resolution and increase escalation risk. Thus, disputes exhibit path-dependent characteristics, where early tensions shape later interactions and generate self-reinforcing conflict trajectories. Moreover, these processes are embedded within broader healthcare governance structures, institutional trust systems, and sociocultural environments, which further complicate prevention and intervention.

Second, while existing studies have identified numerous influencing factors, the current literature predominantly focuses on patient perspectives, public attitudes, legal dimensions, or analysis of medical malpractice cases. In contrast, relatively limited attention has been paid to the physician perspective, particularly a lack of in-depth qualitative exploration of how doctors themselves understand the origin, development, and escalation of medical disputes in daily clinical practice. Cross-national comparative studies in this regard are even scarcer. This research gap is significant because physicians occupy a dominant position in the doctor-patient relationship and serve as core practitioners within the medical system (19, 20). A physician-centered perspective may help reveal the interplay between institutional arrangements, clinical practice environments, communication processes, and dispute formation mechanisms—dynamics that are often difficult to fully capture through patient-centered research approaches alone. China and Japan provide a valuable comparative context for addressing these gaps.

As two East Asian societies, both countries share important cultural foundations, particularly influences from Confucianism and Buddhism, as well as broadly comparable goals regarding healthcare accessibility and population health. Nevertheless, substantial differences exist between the two healthcare systems in terms of institutional design, resource distribution, workforce organization, medical security systems, healthcare financing, and overall quality and accessibility of medical services (21–26). These structural differences may shape levels of institutional trust and the characteristics of medical disputes. Comparative investigation between China and Japan therefore offers an opportunity to better understand the emergence and evolution of medical disputes in different medical practice environments.

Against this background, the present study adopts a qualitative comparative research design to explore the processes by which medical disputes arise and factors influencing their development from perspectives of doctors. The aims of this study are to: (1) identify context-specific factors contributing to medical disputes in China and Japan; (2) elucidate the mechanisms by which these factors operate and construct a causal network of medical disputes; and (3) propose insights to reduce disputes and improve the clinical practice environment.

## 2 Methods

This study is the fourth component of a China–Japan comparative project on DPRs, following three papers in *Asian Bioethics Review* on DPR models, decision-making styles, and doctors' responses to patient death and medical disputes (10, 11, 27). Semi-structured interviews were conducted with 20 Chinese and 20 Japanese doctors, using the same language-specific guide, focusing on causes of medical disputes. This study was approved by the Ethics Committee of Tohoku University Graduate School of Medicine on January 26, 2023 (2022-1-886). Reporting follows the COREQ checklist (28).

### 2.1 Participants

We employed purposive sampling, a standard qualitative approach in which participants are intentionally selected according to study aims and contextual relevance (29, 30). Because doctor–patient relationship (DPR) problems and medical disputes in China are concentrated primarily in university hospitals, whereas Japan generally shows more stable DPRs across diverse institutional settings (2, 7–9), Chinese participants were recruited from settings most closely associated with the phenomenon under investigation, while Japan was included as a contrasting case to facilitate external comparative interpretation beyond a solely China-centered perspective.

Forty physicians from both countries were recruited through researchers' professional networks, with limited snowball sampling used to access clinicians with direct experience relevant to the study topic. All participants had completed clinical training and possessed at least five years of clinical experience. Participation was unpaid.

This recruitment strategy prioritized information-rich and contextually relevant cases rather than statistical representativeness, consistent with qualitative comparative research (29, 31–33). Given the methodological and logistical difficulty of conducting matched or stratified cross-national qualitative sampling in a complex and sensitive research area, one-to-one matching between Chinese and Japanese participants was not attempted.

### 2.2 Data collection

Semi-structured interviews were conducted via Zoom or in person. One participant, unable to join via Zoom, was interviewed face-to-face by A.A. (Atsushi Asai) at his office. Between March and April 2023, all five co-researchers conducted interviews in both countries using these methods.

To minimize cross-linguistic and cross-cultural bias, Yining R (Y.R.), proficient in Chinese, Japanese, and English and familiar with Chinese culture, joined as a translation assistant. Before interviews, the team reviewed literature on DPR issues and developed an exploratory interview guide, originally in Japanese and translated into Chinese by H.X., A.A., and Y.R. After consensus and multiple revisions, the guide was finalized in both languages, ensuring rigor, quality, and applicability.

Before formal interviews, participants were informed of the study objectives and methods. Written informed consent was obtained. Participants were told that interviews would be audio-recorded, anonymized, and kept strictly confidential.

During the interviews, before asking participants about the current situation and causes of medical disputes, we briefly introduced examples of factors related to both physicians and patients as neutral prompts to facilitate discussion on this potentially sensitive topic and to encourage reflection from multiple perspectives, rather than to direct responses toward predefined explanations. Participants were explicitly encouraged to elaborate on, reject, or introduce factors beyond the examples provided. We then asked the following open-ended questions from the interview guide:We then asked them the questions in Interview Outline: “What do you think about the current situation of doctor-patient disputes (all conflicts, wrangling, aggressive attitudes, violence, lawsuits, etc. between patients and medical personnel) in the current medical field?”, “What do you think are the main causes of doctor-patient disputes? When do you think patients and their families become angry or hostile?”, “What do you think of the medical institution”s response to physician-patient disputes when they occur? Is it sufficient or problematic? What are the reasons?” and “How do you think we can prevent doctor-patient disputes in the future? Please share your thoughts.”.

### 2.3 Data analysis

Qualitative descriptive analysis was used to develop subcategories and categories regarding the causes of medical disputes in both countries (34, 35). The following steps were taken.

First, following a “mother tongue first principle” (35), H.X. (XU HUA Hua Xu) and A.A. reviewed the responses to these questions several times to obtain a complete picture of the interviewees' responses. All Chinese and Japanese interviews were first transcribed verbatim and translated by native speakers into Japanese and Chinese, respectively. DeepL was then used primarily to generate parallel Chinese–Japanese–English versions for cross-language comparison and consistency checking. Key meanings and culturally nuanced expressions were further reviewed and refined by the research team during analysis. Meaning units relevant to the study topics were then extracted from the finalized English texts, organized into two Excel files (China and Japan), and systematically coded.

Second, coding followed a joint, iterative consensus-building process under A.A.'s supervision. Meaning units followed by coding of the meaning units in each file. Codes with similar content were grouped into subcategories, and subcategories with common themes or implications were grouped into categories. We continued the same process iteratively and all researchers confirmed and agreed on the appropriateness of all generated codes, subcategories, and categories.

Third, to ensure coding consistency and reliability, initial coding, one of the study's most challenging stages, had minor disagreements between X.H. and A.A. X.H. conducted a consistency check, calculating an initial coding agreement rate of 94%—1 minus the proportion of disputed or controversial codes. Additionally, preliminary category–subcategory findings were returned to participants for validation, with no validity concerns reported.

Last, although no formal saturation assessment was conducted, analysis of 20 Chinese and 20 Japanese interviews revealed no new themes. This is consistent with prior evidence suggesting that 12–30 participants are typically sufficient to achieve theoretical saturation in cross-cultural qualitative research (35–37).

## 3 Results

Table 1 presents the background of participating doctors. Table 2 identifies five categories and seventeen subcategories of medical dispute causes, reflecting views from doctors in both countries. Categories provide a summary of subcategories, while subcategories detail situational and institutional aspects, with supporting quotes included. Numbers in Table 2 serve as analytic markers indicating the frequency and relative salience of viewpoints, without statistical significance.

**Table 1:** Demographic characteristic of the study participants.

Category	China	Japan
	No. or mean ± SD (*N* = 20)	No. or mean ± SD (*N* = 20)
Interview time mean (min)	50	52
Gender		
Female	4	3
Male	16	17
Age		
20's	1	0
30's	4	1
40's	12	5
≧50's	3	14
Working experience	16 ± 8	29 ± 13
5–9 y	4	0
10–19 y	12	2
20–29 y	2	9
≧30 y	2	9
Hospital type		
Primary healthcare institution		5
Secondary hospital		6
University hospital	20	9
Specialty		
Internal medicine	2	14
ER	3	
Surgery	14	6
ICU	1	
Conflict experience with patient sides		
Aggressive attitudes/words	19	2
Physical attack	6	1
Personal/professional involvement in formal dispute process	17	7

**Table 2:** Causes of medical disputes from the opinion of doctors in China and Japan.

Categories (*N* = 5)	Subcategory (*N* = 17)	CD	JD
		No.	No.
Financial issues impact DPRs	Healthcare insurance quality is a critical factor underlying disputes in China and Japan.	18	8
	Low economic level is the main factor underlying disputes in China and Japan.	10	1
	Consumerism has a negative impact on DPRs in China and Japan.	8	2
Medical disputes are inevitable due to poor medical visitation and legal systems	Medical disputes result from the poor medical dispute resolution in China.	16	
	Poor medical consultation systems affect DPRs in China.	6	
	Old-fashioned medical system in university hospitals prevents the quality of medical training and care from improving in Japan.		2
	Strict legal systems cause medical disputes in China and Japan.	4	3
	Patient clustering is a major problem in China and Japan.	7	6
Vicious cycle exacerbates medical disputes in China	Collapse of social morality causes medical disputes in China.	6	
	Poor practice environment increases medical disputes in China.	11	
	Poor compensation practices contribute to creating and perpetuating a vicious cycle in China.	8	
	Disparity in the risk of medical disputes and workload of doctors exists in China.	7	
Distrust results in medical disputes	Mutual distrust causes medical disputes in China and Japan.	12	2
	Distrust is not an issue for the public and patients in Japan.		3
	Social proximity is essential to build mutual trust in China.	4	
	Concealment makes both sides suspicious in China and Japan.	4	8
Poor communication and bad attitude lead to medical disputes	Poor communication and bad attitude lead to medical disputes in China and Japan.	12	17

*“CD” means Chinese doctor, Japanese doctor are represent by “JD”.

### 3.1 Financial issues impact DPRs

In this section, we describe three aspects related to financial issues: “Healthcare insurance quality is a critical factor underlying disputes in China and Japan,” “Low economic level is the main factor underlying disputes in China and Japan,” and “Consumerism has a negative impact DPRs in China and Japan.”.

#### 3.1.1 Healthcare insurance quality is a critical factor underlying disputes in China and Japan

Eighteen Chinese doctors identified inadequate health insurance as a key driver of medical disputes. Due to systemic limitations, patients often face heavy out-of-pocket burdens and express frustration during care. For example, in ENT cases, hemostatic materials can reduce pain but are more expensive than surgery and are not reimbursable, making them inaccessible to most patients. Reimbursement rates also vary by hospital level, with tertiary hospitals often offering lower rates; for instance, treatment for chronic obstructive pulmonary emphysema is reimbursed at about 90% in county-level hospitals but only around 60% in tertiary hospitals. Meanwhile, existing insurance caps do little to deter patients from seeking care for minor conditions at tertiary hospitals, where high-quality doctors are concentrated despite higher costs. Consequently, doctors often spend substantial time explaining insurance coverage and costs, sometimes exceeding the time spent on clinical treatment itself.

“Improving the healthcare insurance system, which can reduce disputes by 70% to 80%, can stop patients from worrying about medical costs.” [CD3].

Eight Japanese doctors noted that Japan's high-quality health insurance system positively supports DPRs by enabling low-cost care and free access. Although some expensive drugs are restricted, elderly patients receive affordable, high-quality care, typically paying only about 10% of monthly treatment costs, with additional support from long-term care insurance.

“Good DPRs in Japan are the result of a comprehensive healthcare insurance system and free access to medical care.” [JD2].

#### 3.1.2 Low economic level is the main factor underlying disputes in China and Japan

Ten Chinese doctors viewed financial constraints as the main driver of medical disputes. Widespread patient poverty limits access to optimal treatments and costly materials, and when outcomes are poor, patients may seek to recover their medical expenses.

“The economy shapes medical disputes. There are still lots of poor people in China. Mainly poor patients sue, while the rich seek treatment abroad.” [CD1].

One Japanese doctor stated that, in Japan, some younger patients are unable to receive medical care due to economic reasons.

“It is still difficult for the younger generation to access healthcare due to economic factors, lack of time, and unstable employment.” [JD9].

#### 3.1.3 Consumerism has a negative impact on DPRs in China and Japan

Eight Chinese doctors argued that consumerism among both patients and doctors negatively affects DPRs. China's medical system pushes hospitals toward commercialization, with market-oriented management and income quotas for doctors. Doctors must complete clinical work while meeting revenue targets. Meanwhile, patients act as consumers purchasing medical services and choosing doctors. If doctors fail to meet patients' expectations, dissatisfaction arises and medical disputes become more likely.

“Patients have unusual expectations of medical care; when doctors fail to meet them, they complain, like consumer-rights claims. They assume medical costs should match outcomes. This consumer model has long existed in the domestic healthcare system. Unless this consumerist mindset changes, doctor–patient relationships will not improve.” [CD7].

Two Japanese doctors noted that patients with a consumer mindset expect instant cures like purchasing services in a shop, and may refuse payment if their expectations are not quickly met or symptoms do not improve.

“Patients expect to receive the same service as they would in a convenience store. They hope the hospital will respond quickly to their needs.” [JD17].

### 3.2 Medical disputes are inevitable due to poor medical visitation and legal systems

In this section, we describe five aspects related to the poor medical and legal systems in both countries: “Medical disputes result from the poor medical system and illegal handling of medical disputes in China,” “Poor medical visit system affects DPRs in China,” “Old-fashioned medical system within university hospitals prevents the quality of medical training and care from improving in Japan,” “Strict legal systems cause medical disputes in China and Japan,” and “Patient clustering is a major problem in China and Japan.”.

#### 3.2.1 Medical disputes result from the poor dispute resolution system in China

Sixteen Chinese doctors believed weak legal compliance fuels medical disputes in China. First, the legal framework and standards for handling disputes are incomplete. Second, under pressure from the public and government, hospitals often take a weak stance and provide illegal compensation to maintain stability, reinforcing the belief that disputes lead to payment. Third, laws also fail to deter patients' illegal behavior due to weak enforcement. Fourth, doctors' rights—such as personal safety, life, privacy, and reputation—are inadequately protected, allowing disputes to persist for decades.

“The lax legal system has led to decades of medical disputes because relevant laws and regulations are incomplete and outdated. Hospitals remain weak in handling disputes and often compensate patients regardless of whether medical errors occurred, which further encourages disputes.” [CD15].

#### 3.2.2 Poor medical visit system affect DPRs in China

Six Chinese doctors stated that inefficient hospital management and poor equipment lead to weak medical visit systems. Cumbersome, inconvenient procedures—such as delayed surgeries, slow lab reports, and poor guidance—worsen patient experience regardless of illness severity. Inefficient processes increase doctors' workloads, while outdated or malfunctioning equipment can delay emergency treatment. These problems reduce care quality, trigger complaints, and sometimes cause patients to vent frustration on doctors.

“The process of visiting a doctor is too cumbersome and inconvenient, which increases the irritability of patients. Sometimes I see several poor patients from rural areas waiting at the hospital gate after the doctor has left work. They had to wait for the hospital to reopen to get their lab reports.” [CD9].

#### 3.2.3 Old-fashioned medical system in university hospitals prevents the quality of medical training and care from improving in Japan

Two Japanese doctors identified *Ikyoku*, an informal university-based medical network linking department members, university hospital supervisors, and affiliated community doctors, as a root cause of problematic DPRs affecting both doctors and patients. Originating in the Meiji era, it prioritizes clinical skill training while monopolizing education, practice, and research, with little emphasis on patient communication. This structure hampers reforms in medical education and patient-centered care, and—because university doctors often hold multiple jobs due to low salaries and frequently attend academic conferences—they are unable to see patients promptly.

“I still think the root of the problem is the Ikyoku system. If this Yakuza (organized crime groups in Japan)-like system in Japan is not changed, patient-centered medicine will be a fantasy.” [JD1].

#### 3.2.4 Strict legal systems cause medical disputes in China and Japan

Four Chinese doctors expressed the view that the medical process is always judged critically by picky medical experts. First, the appropriateness of medicine is judged from the point of view of results rather than medical guidelines. Second, medical experts become very critical and caustic in identifying minor medical problems. In fact, in most cases there are no fundamental errors. Third, strict adherence to treatment guidelines is unrealistic for doctors in actual clinical practice.

“Do not use the standard of a saint to ask a doctor. So, I believe the law should provide doctors with a mechanism that allows for mistakes. Should doctors be permitted to make mistakes when dealing with human life?” [CD20].

Three Japanese doctors stated that Japan's strict legal system conflicts with clinical practice. Criminal law is applied to medicine, legal and medical cultures differ, some lawyers expand disputes for compensation, and unreliable treatment guidelines constrain doctors, increasing medical disputes and worsening DPRs.

“A doctor should not be penalized because a bad outcome is not a deliberate medical error.” [JD7].

#### 3.2.5 Patient clustering is a major problem in China and Japan

Seven Chinese doctors believed that at least 30% of medical disputes in China stem from patient clustering. Causes include poor triage systems, unequal distribution of medical resources concentrating skilled doctors and equipment in large hospitals, and patients' poor medical understanding, leading even terminal patients to crowd ICUs. This creates chaotic environments, limited doctor–patient communication, poor experiences, wasted resources, and worsened DPRs.

“I saw more than 40 patients on night shift, mostly clustered at dawn; I treated them while maintaining order and preventing fights.” [CD17].

Six Japanese doctors noted that although healthcare quality and doctors' skills are similar across institutions in Japan, patient clustering still occurs. There are three factors contribute to this: low medical costs and free access under the insurance system, along with lucrative reimbursements that fail to limit patient numbers and tests; weak appointment systems allowing walk-in visits to specialists; and inexpensive specialist access. These factors cause patient concentration, shortening consultation time and reducing the quality of care for each patient.

“Thanks to the reimbursement system, patients can visit doctors freely, but the lack of limits increases patient numbers, shortens consultation times, and results in poorer medical service for each individual patient.” [JD15].

### 3.3 Vicious cycle exacerbates medical disputes in China

Chinese doctors noted a vicious cycle in clinical practice that is created by a negative social environment, poor medical environment, mass media problems, bad habits in terms of remuneration, and the inequality between risk and workload among doctors. These problems interact and aggravate each other, creating a vicious cycle.

In this section, we discuss four aspects which relate to this vicious cycle: “Collapse of social morality causes medical disputes in China,” “Poor practice environment increases medical disputes in China,” “Poor compensation practices contribute to creating and perpetuating a vicious cycle in China,” and “Disparities in the risk of medical disputes and workload of doctors exist in China.”.

#### 3.3.1 Collapse of social morality causes medical disputes in China

Six Chinese doctors argued that poor DPRs reflect broader social problems in China and a deteriorating public spirit. They attribute medical disputes mainly to declining social morality in three aspects: the mismatch between wealth, morality, and social status; widespread social distrust caused by economic development and dishonesty across industries; and the resulting erosion of social morality and human virtue in a negative social environment.

“Medical disputes reflect greed from socioeconomic development. China's poor social environment weakens DPRs and social trust, while Japan performs better across food safety, healthcare, and social integrity.” [CD8].

#### 3.3.2 Poor practice environment increases medical disputes in China

Eleven Chinese doctors noted that rising medical disputes and deteriorating DPRs have worsened the medical environment, affecting hospital management and doctors' behavior and psychology. Under operational pressure and dispute risks, hospitals shift from professionalism to emphasizing service attitude, neglecting technological advancement. This leads to stagnating technical levels, poor talent cultivation, dishonest practices, and weakened safety culture. Doctors may focus on superficially maintaining relationships and avoiding disputes rather than patient care. Fear of punishment also makes doctors avoid difficult treatments and reduces motivation, ultimately harming medical professionalism and patient care.

“In the daily department meetings, the idea conveyed by the department leaders is that one must find a way to eliminate risks for oneself. This kind of thinking has been instilled over time, making doctors afraid to work and unable to treat patients with ease.” [CD18].

#### 3.3.3 Poor compensation practices contribute to creating and perpetuating a vicious cycle in China

Eight Chinese doctors expressed the view that the media and Internet are one-sided and exaggerate the reporting of medical disputes, and their claims contribute to social misunderstanding and hostility towards medical professionals. The presumption that doctors are always wrong when it comes to medical disputes exacerbates DPRs. Coupled with pressure from the government, hospitals often pay compensation even when there have been no errors. This has led hospitals to adopt a bad habit of using compensation to settle disputes, regardless of whether there is a medical error or not. The public's mistaken belief that they can get a payout from medical disputes increases the number of disputes. The habit of compensating patients contributes to the creation and perpetuation of a vicious cycle.

“When it comes to disputes between doctors and patients, the public first condemns the doctor. Now real and fake video information is overwhelming and everyone can search it. Such negative and heavy pressure forces the hospital to settle disputes as soon as possible through compensation. It is true that compensation is for the sake of peace.” [CD16].

#### 3.3.4 Disparity in the risk of medical disputes and workload of doctors in China

Seven Chinese doctors believed that in poor clinical settings, frequent disputes and violence make doctors nervous and fearful, causing them to view their work as risky and burdensome. They perceive an imbalance between dispute risk and workload: more work leads to more mistakes and disputes; disputes bring punishment, criticism, and reduced income; and doctors become afraid to work as before. Moreover, income is not clearly linked to workload. These factors weaken doctors' enthusiasm, hinder skill improvement, encourage defensive medicine, and create a vicious cycle with patient dissatisfaction.

“Doing less can be safer and cause less trouble and conflict. The hospital should clarify and improve the system of rewards and punishments so that doctors can continue to work normally after a dispute without fear of punishment or making mistakes.” [CD14].

### 3.4 Distrust results in medical disputes

In this section, we discuss four aspects which relate to the problem of distrust: “Mutual distrust causes medical disputes in China and Japan,” “Distrust is not an issue for the public and patients in Japan,” “Social proximity is essential to build mutual trust in China,” and “Concealment makes both sides suspicious in China and Japan.”.

#### 3.4.1 Mutual distrust causes medical disputes in China and Japan

Twelve Chinese doctors believe medical disputes stem mainly from mutual distrust. Patients sometimes trust online medical information more than doctors, increasing stress and fostering defensive attitudes on both sides. Distrust leads patients to demand care their way, while doctors may resort to defensive medicine, including over-testing or withholding treatment details. This raises patient costs, prompting blame if tests show no illness, further fueling dissatisfaction and distrust.

“Originally, every doctor is kind and tries hard to cure the disease, not to deceive. There is no such case of deceiving patients. The patient's factors are what cause the doctor's deceptive behavior.” [CD12].

Two Japanese doctors expressed the view that a lack of trust can lead to litigation or violence. One Japanese doctor described the presence of a patient who stated that he could not accept a situation where the doctor who explained the operation to him and the actual surgeon differed, or where he did not know who would be performing the operation until the day of the operation.

“Good DPRs are built on trust. Trust can prevent lawsuits. When trust is lost, the DPR deteriorates, and even minor issues can lead to litigation.” [JD18].

#### 3.4.2 Distrust is not an issue for the public and patients in Japan

Three Japanese doctors stated that distrust is likely no longer an issue in Japan, as the public generally trusts doctors due to positive media portrayal of the medical system.

“I don't think there are many people who come to a hospital with a sense of distrust from the start. But when the result is bad, I think all the things that have built up over the years come to the surface and become apparent.” [JD14].

#### 3.4.3 Social proximity is essential to build mutual trust in China

Four Chinese doctors noted that personal closeness is crucial in medical treatment. Patients prioritize finding doctors via social connections over technical skill, building trust through acquaintances or red envelopes (gifts), though giving/accepting red envelopes is now deemed unethical. Banning this practice undermines trust, and patients' preference for familiar doctors creates heavy psychological pressure on physicians.

“Chinese patients often rely on social connections and familiar doctors, sticking with them long-term. Consulting other doctors via relatives to verify treatment can make the treating doctor feel that the patient does not trust him, increasing their mental stress.” [CD19].

#### 3.4.4 Concealment makes both sides suspicious in China and Japan

Four Chinese doctors identified four types of concealment: first, doctors may inaccurately describe procedures or falsify reports; second, hospitals may hide treatment errors and give patients false explanations; third, even if the doctor is not at fault, hospitals may suppress correct information, forcing doctors to admit mistakes and compensate patients; fourth, some patients are dishonest, denying facts and blaming doctors for poor outcomes.

“Sometimes the explanation of treatment given by doctors is falsified. There are many disputes arising from mistakes in the medical process. We are also shocked when we hear the truth about the operation process. In the future, the medical industry will get worse and worse.” [CD13].

Eight Japanese doctors noted that while many hospitals respond honestly to disputes, some doctors and institutions conceal information and act self-servingly, undermining patient trust and causing disputes.

“The doctor behaves dishonestly, which destroys trust.” [JD12].

### 3.5 Poor communication and bad attitude lead to medical disputes in China and Japan

Twelve Chinese doctors expressed the view that medical disputes can be caused by poor communication and inadequate informed consent. They provided four main reasons for poor communication, in order of importance: time limitation, poor communication skills, and inadequate IC, as well as bad attitude of both sides.

“Doctors also would like to communicate more and treat patients well, but time constraints and numerous other tasks make this difficult.” [CD4].

Seventeen Japanese doctors expressed the same view that most disputes are caused by poor communication skills, bad attitude of both sides, and insufficient explanation, time limitation, as well inadequate informed consent.

“Lack of communication is a major issue. Medical care revolves around people and communication. Doctors should first consider the reasons behind patients' words and their main concerns, instead of reacting immediately, to make patients feel cared for.” [JD11].

## 4 Discussion

Chinese and Japanese participants revealed diverse factors influencing the occurrence of medical disputes within the same overarching categories and subcategories (Objective 1). Beyond factor identification, participants also provided rich contextual accounts elucidating how and why these factors operate in practice. As this discussion is context-specific and limited to the Chinese and Japanese medical environments examined in this research, the following analysis focuses on the mechanisms through which key factors contribute to medical disputes and integrates them into a causal network (Objective 2), thereby laying the foundation for the dispute-reduction strategies presented in the Conclusion (Objective 3). Specifically, the Discussion examines financial issues (4.1), healthcare consumerism (4.2), deficiencies in medical dispute resolution systems (4.3), and issues of distrust (4.4), before synthesizing these elements into a comprehensive causal network of medical disputes (4.5).

### 4.1 Financial issues: patient poverty and health insurance quality

Chinese participants repeatedly emphasized that widespread patient poverty and the limited protective capacity of the health insurance system jointly generate a heavy medical cost burden. In contrast, Japanese participants rarely mentioned poverty and frequently highlighted the high quality of Japan's health insurance system as a key institutional foundation supporting stable DPRs. Taken together, these findings suggest that financial conditions—particularly patient poverty and insurance quality—operate as important antecedent factors influencing the emergence of medical disputes.

#### 4.1.1 Structural context of financial vulnerability

Patient poverty in China is embedded in broader structural and historical contexts. Poverty is inherently relative and shaped not only by material deprivation but also by individuals' lived experiences and perceptions (38).

Compared with Japan, China's modernization began from a significantly lower economic base and continues to face challenges associated with a large population and uneven regional development. Although rapid economic growth since the reform and opening-up period has lifted large numbers of people out of absolute poverty, economic vulnerability remains widespread. Average income levels remain substantially lower than those in Japan, while the share of household expenditure devoted to healthcare is considerably higher (39).

Perceptions of financial hardship are closely related to the strength of social protection systems. In China, households continue to face significant financial pressure from the widely recognized “three mountains” of housing, education, and healthcare or elderly care (40). By the end of 2021, expenditures related to medical care, education debt, housing loans, and educational spending had risen to levels exceeding the OECD average (41). Although economic growth and urbanization have reduced absolute poverty, new forms of vulnerability persist, particularly among rural residents, older adults, and left-behind children with limited access to stable income or social security (42). In contrast, Japan established a comprehensive social security system as early as the 1960s (43), enabling households to better absorb financial shocks associated with illness, unemployment, or aging. As a result, poverty remains a more salient social condition shaping medical experiences in China.

Health insurance systems further structure this financial vulnerability. Both China and Japan have achieved universal health insurance coverage, but the depth and effectiveness of coverage differ substantially. Although China's system covers most of the population, reimbursement levels remain limited, and out-of-pocket expenditures are significantly higher than those in Japan (44, 45). No healthcare system in the world is perfect, Japan also faces a range of significant challenges, such as the immense pressure of an aging population on its healthcare and pension systems,widespread hospital operating losses, a shortage of medical personnel, rising medical costs (44). However, in this study, Japanese participants frequently emphasized that Japan's insurance system provides relatively low costs and high-quality care, particularly benefiting older adults.

By contrast, Chinese participants highlighted several institutional limitations within China's health insurance system. These include disparities across regions and insurance schemes, higher reimbursement rates for employee-based insurance compared with schemes covering children, older adults, migrant workers, and unemployed individuals, and persistent problems of under-coverage or unstable enrollment among vulnerable populations (46). Although China introduced outpatient reimbursement coverage in 2023, many restrictions remain, leaving patients responsible for a substantial proportion of medical costs—often 50% to nearly 100%, compared with roughly 30% in Japan (47, 48). The limited scope of reimbursable drugs and medical materials further increases the financial burden, especially for patients with chronic conditions.

#### 4.1.2 Mechanisms linking financial pressure to medical disputes

Within this structural context, financial pressures influence DPRs and contribute to medical disputes through several interconnected mechanisms.

First, poverty directly constrains patients' ability to access recommended medical care. Financial hardship may prevent patients from selecting optimal treatment plans, completing necessary examinations, or purchasing expensive medical materials recommended by physicians. For economically vulnerable patients, basic subsistence often takes precedence over healthcare spending. This dynamic reinforces the well-known cycle in which poverty exacerbates illness while illness further deepens poverty (38). When treatment is discontinued because of financial constraints or when clinical outcomes are unfavorable, responsibility is often attributed to doctors or hospitals, creating conditions for conflict.

Second, financial stress influences patients' expectations and behavioral responses toward medical care. Participants reported that economically disadvantaged patients are more likely to adopt a consumerist understanding of medical services, hold unrealistic expectations regarding treatment outcomes, and display limited tolerance for medical risks (49). When outcomes fail to meet expectations, financial anxiety can easily transform into dissatisfaction, complaints, or demands for compensation. Previous studies likewise indicate that medical disputes occur more frequently among socially and economically disadvantaged groups, who often have limited insurance protection and may experience stronger feelings of neglect or relative deprivation during clinical encounters (50, 51).

Third, institutional constraints within the health insurance system further intensify financial tensions during clinical interactions. Chinese participants noted that restrictive reimbursement policies require doctors to spend considerable time explaining treatment costs, and many disputes arise not from medical error but from patients' inability to afford out-of-pocket expenses. When medical bills become overwhelming, families may experience severe financial distress that is redirected toward doctors and hospitals in the form of blame or confrontation (10). Participants also observed that the limited scope of reimbursable drugs may force patients to accept less effective or more burdensome treatment options, generating dissatisfaction that further undermines trust.

Finally, financial pressures interact with broader characteristics of the healthcare system to exacerbate tensions within DPRs. For example, patient clustering in large hospitals—partly linked to uneven distribution of medical resources—creates overcrowded clinical environments and severely limits communication time between doctors and patients (52). Under such conditions, financial stress, institutional constraints, and limited communication often converge, increasing the likelihood that DPRs become defensive or confrontational.

In summary, financial issues constitute a fundamental structural antecedent shaping DPRs and the occurrence of medical disputes. In China, the combination of persistent economic vulnerability and limited insurance protection generates chronic financial uncertainty for patients, which erodes trust and intensifies conflict during clinical encounters. By contrast, Japan's high-quality health insurance system reduces economic uncertainty and provides an institutional foundation for more stable and cooperative DPRs.

### 4.2 Impact of healthcare consumerism on medical disputes

Many Chinese doctors emphasized that consumerism has long been embedded in China's healthcare system and constitutes a primary structural factor underlying medical disputes—a view rarely mentioned by Japanese participants. Accordingly, consumerism is regarded as an important antecedent shaping contemporary DPRs. This section examines healthcare consumerism at two levels: first, the institutional background of healthcare consumerism in China, and second, the structural mechanisms by which consumerism contributes to medical disputes within this context.

#### 4.2.1 Institutional background of healthcare consumerism in China

Healthcare consumerism positions patients as consumers and doctors as service providers. Many Chinese participants noted that China's medical system has long pressured public hospitals to operate in a market-oriented manner, with explicit task quotas and revenue targets for doctors. Under these conditions, a consumerist healthcare model was formed and has persisted for decades, rooted in medical reforms reinforcing distortions in healthcare.

Since the 1980s, China's healthcare delivery system has undergone profound marketization alongside healthcare reforms and the broader reform-and-opening-up policy (53). Public hospitals widely adopted a “self-financing and profit-and-loss responsibility” model, under which doctors' incomes were closely linked to departmental and individual revenue generation. Hospitals thus occupy a dual role as both prescribers and sellers of drugs (27).

Over-commercialized medicine has fueled a self-reinforcing cycle of healthcare consumerism and deepening distrust in DPRs for more than three decades (54). For decades, doctors and hospitals have derived significant benefits from the sales of pharmaceuticals and medical devices, with drug revenues and testing fees remaining the two primary sources of income for public hospitals (21, 55). Consequently, DPRs have, in practice, shifted toward interest-based interactions and, in some cases, have been perceived as explicit consumer relationships (56). By contrast, Japan's post-1980 reforms retained a non-profit healthcare system, in which public subsidies and the separation of prescribing and dispensing constrain conflicts of interest and sustain system-level doctor–patient trust (44).

China has implemented policies since 2012 to curb pharmaceutical rebates, culminating in a formal ban by the NHSA in October 2023. Yet, after over a decade, the effectiveness of these reforms remains limited due to the absence of compensating hospital funding mechanisms and rational doctor remuneration systems (57). Hospitals reliant on drug and testing revenues faced heightened financial pressure, partly transferred onto doctors. The resulting reduction in doctors' income undermined their motivation and commitment. Under these compounded structural constraints, doctors developed a profound sense of imbalance between income, workload, and medico-legal risks. This perceived unfairness forms a critical link in the vicious cycle—shown in Table 2—where a deteriorating medical environment, worsening DPRs, and escalating disputes reinforce each other.

#### 4.2.2 Mechanisms by which consumerism shapes the occurrence of medical disputes

Chinese participants pointed out that consumerism in healthcare has gradually eroded doctors' clinical decision-making and professional morality, inflated patients' unrealistic expectations, and ultimately undermined doctor–patient trust.

Chinese participants noted that decades of excessive medical commercialization in China have inevitably transformed DPRs into interest-based or explicitly consumer-oriented interactions. In this process, the traditional trust-based DPR—conceived as a trust-grounded community centered on patients' interests—eroded substantially, as the consumer-oriented DPR instead prioritizes the interests of doctors and medical institutions (17). Beyond low-quality health insurance (4.2), the commercialization of healthcare increases the financial burden on patients and erodes trust. In large Chinese hospitals, profit-driven use of discounted drugs, high-end equipment, and excessive testing further inflates costs, with many patients perceiving overprescription and calling for more reasonable examinations (58).

Chinese participants further emphasized that the commercialization of healthcare has fostered a consumerist mindset among patients. Medical consumerism has not only altered doctors' clinical decision-making but also profoundly reshaped patients' healthcare-seeking attitudes. They also claimed that patients increasingly evaluate medical services from a “consumer” standpoint, and unmet expectations are more likely to lead to compensation-oriented disputes. Many patients equate “paying for treatment” with consumption, believing that if doctors fail to cure the illness, they should “compensate with money—or even with their lives.”.

Chinese participants also noted that, under sustained operational pressure and the rising risk of medical disputes, many large hospitals have gradually shifted their focus from professionalism to improving service attitude. This shift has generated new issues relating to doctor–patient trust, including dishonest behavior by doctors and the erosion of hospital safety culture. Some Chinese participants described this reality: many doctors prioritize displays of warmth and politeness over improving clinical competence to gain patient trust and avoid disputes. As laypersons, patients often perceive only the doctor's attitude, not clinical competence, and may remain trusting even when treatment fails.

In summary, embedding market logic into healthcare in China has led to alienation of the DPR, a crisis of medical professionalism, distortions in clinical practice, and the erosion of trust. These problems do not stem simply from moral decline at the individual level, but reflect entrenched historical medical reforms and constraints in systems.

### 4.3 Medical disputes result from the poor legal system for medical dispute resolution in China

Sixteen Chinese participants identified the poor system of medical dispute resolution as a key factor contributing to the occurrence of medical disputes—a perspective not raised by Japanese participants. This section treats deficiencies in the medical dispute resolution system as a key antecedent shaping contemporary DPRs. It examines two levels: first, the system's operation in practice; and second, with this context, how the system's flaws shape DPRs and exacerbate medical disputes.

#### 4.3.1 Practical operation of China's medical dispute resolution system

China is currently in a transitional and experimental phase in reforming its medical dispute resolution mechanisms. Although a diversified dispute resolution framework with localized characteristics has been preliminarily established, its practical operation continues to reveal significant structural deficiencies (2, 59).

Drawing on international experience—particularly Japan's settlement-oriented approach and third-party mediation mechanisms—China has, with the Regulation on the Prevention and Handling of Medical Disputes enacted in 2018 as its core, formally institutionalized multiple channels for resolving medical disputes (2). These include direct negotiation between parties, people's mediation, administrative mediation, judicial litigation, and other legally prescribed pathways, with people's mediation positioned as the primary mechanism.

Against the backdrop of a high incidence of medical disputes and an underdeveloped legal environment, China's multi-channel dispute resolution system has frequently malfunctioned in practice (60). Existing legal arrangements have failed to provide efficient, fair, and transparent resolution pathways for either party. This institutional weakness has, in effect, incentivized patients to pursue rapid compensation through disruptive behaviors, media pressure, or even violence, creating an environment of “poor compensation practices” (Table 2). By contrast, Japan relies on mature third-party investigation and mediation mechanisms, comprehensive medical liability insurance and no-fault compensation schemes, and institutionalized medical error disclosure, enabling disputes to be resolved within rational and professional boundaries, with violent conflict remaining exceedingly rare (61).

There are also marked differences between China and Japan in regulating doctor–patient behavior and the severity of legal sanctions. China has only recently sought to curb patient violence by criminal law, whereas Japan, through a more refined medical–legal framework that carefully integrates criminal law into medical regulation, has avoided the vicious cycle in which legal intervention itself exacerbates doctor–patient conflict, achieving a relatively stable institutional balance among patient rights, doctor protection, and medical safety (10).

Finally, a deep-seated distrust of law is widespread in Chinese society. Rooted in historical and cultural traditions and reinforced by dissatisfaction with the law, this distrust profoundly undermines the resolution of medical disputes.

#### 4.3.2 Inadequacy of the medical dispute resolution system in contributing to a reduction in medical disputes

Sixteen Chinese participants noted that structural deficiencies in the legal system for medical dispute resolution have weakened medical dispute governance and fostered adversarial and illegal practices, thereby undermining legal compensation practice and clinical working environments and eroding trust in DPRs.

Most Chinese participants pointed out that excessive intervention by government authorities and public opinion, combined with an underdeveloped legal framework for medical dispute resolution, generates structural unfairness in dispute handling and encourages an adversarial, non-institutionalized mode of resolution. Under pressure from both the public and the government, hospitals often take a weak stance towards patients and resort to illegal or informal compensation to maintain peace. This practice entrenches the social expectation that “disruption leads to compensation,” thereby normalizing a distorted and dysfunctional compensation practice (59).

Many Chinese participants also noted that medical experts often conduct peer reviews with a “fault-seeking” orientation, scrutinizing clinical details excessively to establish grounds for compensation claims, even though fundamental errors are lacking in most cases. This tendency to “find faults for the sake of fault-finding” worsens the medical practice environment. China's current medical laws have gaps, loopholes, and ambiguities in key areas like medical process evaluation and negligence determination, leading to arbitrary and informal dispute resolution. For instance, provisions on informed consent are broad and poorly defined, frequently resulting in liability and compensation claims for “insufficient informed consent,” a trend particularly pronounced in China (60).

In addition, existing medical laws fail to effectively constrain and sanction doctors' dishonest conduct. Chinese participants described broader scenarios of concealment involving doctors, patients, and hospitals. One typical scenario is that some medical institutions and doctors conceal medical errors or fabricate or alter medical records. Such practices not only directly infringe on the legitimate rights of patients but also fundamentally erode medical professionalism and affect foundations of trust in DPRs.

Finally, Chinese participants noted that existing laws remain limited in their deterrent effect against patient violence, leaving doctors' personal safety, right to life, privacy, and reputation insufficiently protected. In recent years, violence against doctors has become a serious issue; its high prevalence has inflicted substantial psychological and physical harm on doctors and undermined their professional dignity (11). Violence in clinical settings fundamentally erodes doctor–patient trust and further distorts clinical decision-making. To avoid perceived risks, some doctors adopt defensive medical practices, which may compromise patients' best interests and drive up medical costs.

In summary, inadequacy of the legal system governing medical dispute resolution in China, together with excessive governmental and media intervention, has weakened the system's legitimacy and regulatory authority across multiple dimensions. These deficiencies undermine the shared interests of doctors and patients and exacerbate systemic distrust within the medical system.

### 4.4 Impact of distrust on medical disputes

Most Chinese participants identified pervasive doctor–patient distrust as the principal driver of frequent medical disputes, whereas Japanese participants emphasized trust as foundational to DPRs. In China, distrust is deeply embedded in clinical practice and shaped by intertwined structural and cultural factors.

Structurally, as discussed above, an unfavorable social–systemic environment—including poverty, limited health insurance coverage, healthcare consumerism, flawed dispute-resolution mechanisms, and declining social morality—creates adverse conditions for medical practice. These factors generate secondary harms to trust, such as financial strain, poor communication, defensive medicine, distorted compensation expectations, and deteriorating medical ethics, further intensified by negative media narratives.

Culturally, trust historically developed within an agrarian “acquaintance society,” where interpersonal proximity and guanxi often substitute for institutional trust. Patients may rely on personal connections or informal practices such as “red envelopes” to navigate systemic barriers. However, proximity-based trust is inherently fragile: it inflates expectations, bypasses formal procedures, and can quickly deteriorate into conflict when outcomes fall short (10). Consequently, distrust has become a defining feature of contemporary DPRs in China. While improving communication remains a cost-effective strategy for preserving existing trust and preventing further deterioration, it is insufficient to repair relationships where trust has already severely eroded or collapsed.

### 4.5 Causal network of medical disputes

**Figure 1:**
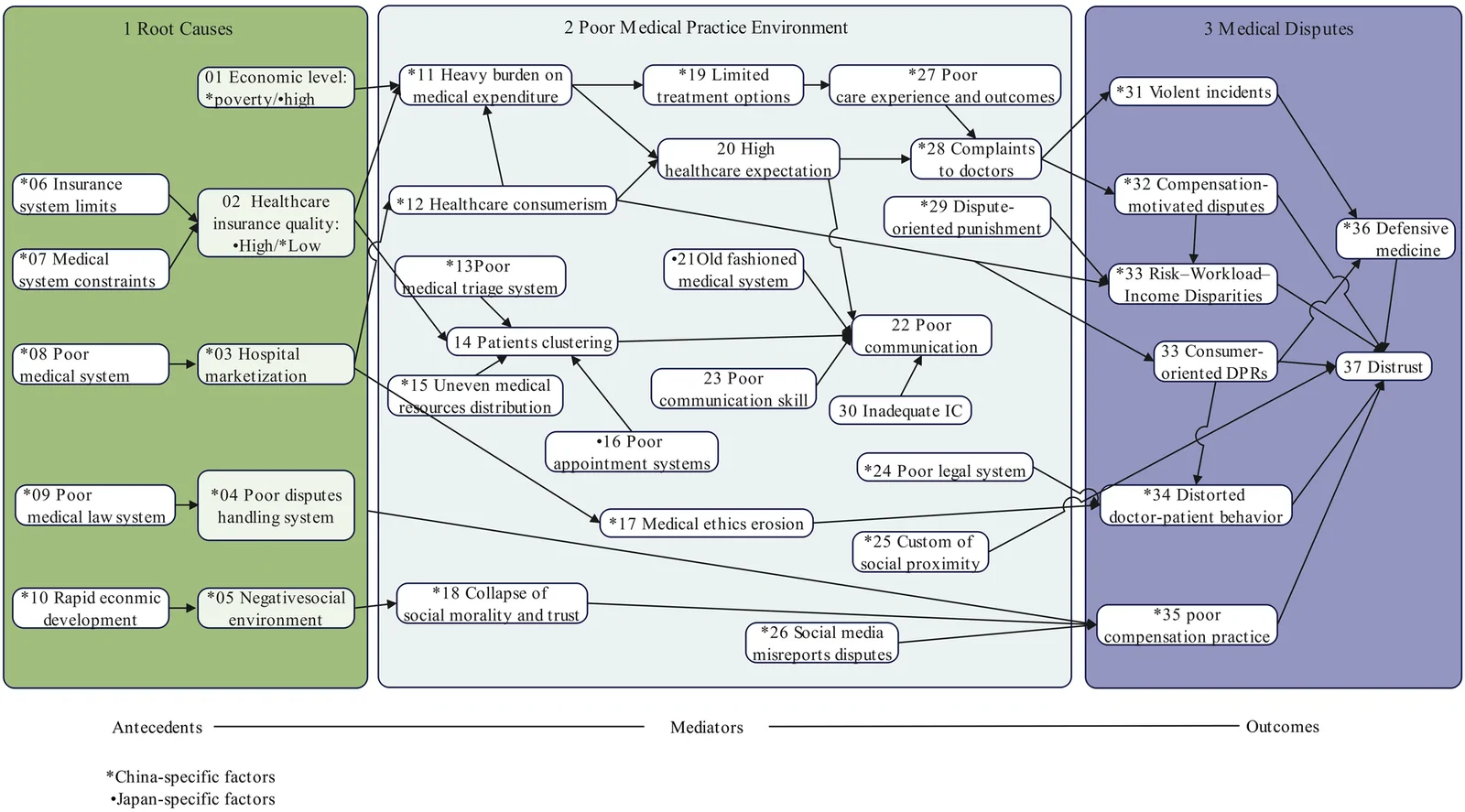
A causal network of medical disputes.

This section presents a conceptual causal network of medical disputes based on the empirical findings of this study. All factors illustrated in Figure 1 were derived inductively from the interview data and are systematically organized into antecedent variables, mediating conditions, and dispute-related outcomes. Rather than functioning as an isolated conceptual diagram, Figure 1 serves as an integrative synthesis of the Results and Discussion sections, bringing together the major themes and relational patterns identified throughout the analysis while preparing the transition toward the Conclusion. Accordingly, the figure should be interpreted in conjunction with the preceding empirical findings and analytical discussions, rather than as a standalone explanatory framework.

Figure 1 illustrates a comparative causal network structured into three analytically distinct yet dynamically interconnected domains: root causes (antecedents), mediating conditions (practice environment), and dispute-related outcomes. The model incorporates both China-specific (asterisked) and Japan-specific (bullet-pointed) factors, thereby highlighting cross-national commonalities alongside context-sensitive differences.

The directional pathways presented in this model were not theoretically predetermined or statistically inferred. Instead, they were inductively derived from physicians' narratives during thematic analysis. During coding and category development, interview data were repeatedly compared across cases to identify recurrent patterns of perceived influence, interaction, and sequential relationships among factors contributing to medical disputes and deterioration of DPRs. Therefore, the relationships illustrated in Figure 1 should be understood as interpretive and perceived relational pathways emerging from qualitative data, rather than objectively verified causal mechanisms. Accordingly, the model is intended as a conceptual and exploratory framework grounded in physicians' experiences.

Poverty (01), low-quality health insurance (02), and market-oriented hospital management (03) constitute a China-specific cluster of antecedent variables that contribute to medical disputes by activating a series of mediating mechanisms. These factors directly impose a heavy medical cost burden (11) on patients, which in turn constrains their ability to make optimal treatment choices (19). At the same time, marketized healthcare operations generate elevated patient expectations (20). When combined with unethical medical practices (17) arising from a commercialized medical environment, patient dissatisfaction (27) is further intensified, and expectations are amplified once again. Under such conditions, even when doctor–patient communication is adequate, the occurrence of an adverse medical outcome (26) can readily trigger compensation-oriented disputes (31) and may escalate into violences on doctors (30). In contrast, this poverty–low insurance–commercialization-driven causal chain is absent in Japan. Instead, a high economic level (02) and high insurance quality (03) function primarily as positive factors that facilitate DPRs.

A second set of antecedent variables in China consists of a poor dispute-handling system (04) and a negative social environment (05), which together generate another causal pathway leading to medical disputes. Within a broader context characterized by a negative social–moral environment (18), and compounded by underdeveloped dispute-handling mechanisms (04) as well as sustained pressure from the government and public opinion (25), medical disputes have long lacked institutionalized resolution. Over time, this has fostered a norm of violent dispute resolution (30), with disputes frequently culminating in hospital compensation (31). This causal pathway—associated with a negative social environment (05) and deficiencies in dispute-handling mechanisms (04)—is likewise not observed in Japan.

Although participants of both countries acknowledged that several mediating variables, such as patient clustering (14), high healthcare expectations (20), and poor communication (24), affect the occurrence of disputes, their antecedent drivers and downstream outcomes differ markedly. For example, some Japanese participants noted that high insurance quality (02) combined with a poor appointment system (16) can lead to patient clustering (14), whereas in China this phenomenon arises from a broader and more complex set of factors, including the low quality of the insurance system (02), limited insurance coverage (06), constraints within the medical system (07), a poorly developed medical triage system (13), and the uneven distribution of medical resources (15). Consequently, patient concentration is more severe in China, and the resulting state of problematic communications (22) is correspondingly deeper. This demonstrates that the same mediating variables intervene through distinct pathways and exert different magnitudes of impact in the dispute mechanisms of both countries.

Taken together, the determinants of medical disputes were perceived by participants to evolve along relatively coherent and interconnected relational pathways. Compared with Japan, China exhibits a substantially larger number of variables across all three categories, resulting in a more complex conceptual causal structure. This structural disparity may help explain the higher incidence of medical disputes in China. Given that the underlying mechanisms and contextual pathways differ between the two countries, their corresponding response strategies are also likely to diverge.

### 4.6 Limitations

This study has several limitations inherent to qualitative and cross-cultural research design.

First, researcher subjectivity may have influenced data collection and interpretation. Four of the five researchers had clinical experience, and one had direct experience handling medical disputes. Although such backgrounds facilitated contextual understanding, they may also have shaped assumptions during interviews and analysis.

Second, the study is subject to sample and representativeness limitations. Purposive sampling was used to select physicians from China and Japan to explore contrasting contexts of doctor–patient relationships (DPRs) and medical disputes. However, the Chinese and Japanese samples were not institutionally or demographically matched, and differences in healthcare systems, institutional structures, specialties, and professional experiences may have affected cross-national comparability. In addition, the relatively small qualitative sample limits the generalizability of the findings.

Third, the study relied exclusively on physicians' perspectives. The absence of patient voices and other healthcare stakeholders may have resulted in partial interpretations of DPRs and medical disputes. Physicians' accounts may also reflect institutional or professional power positions, potentially shaping how conflicts and responsibilities were interpreted. Furthermore, the study did not employ methodological triangulation through additional data sources such as observations, policy documents, or quantitative data, which may limit the comprehensiveness and confirmability of the findings.

Fourth, several methodological limitations relating to data collection and analysis should be acknowledged. Interviewers did not receive formal training, and pilot interviews were not conducted prior to data collection, which may have affected interview consistency and depth. In addition, although initial inter-coder agreement was assessed, independent double-coding and repeated coder reliability checks were not systematically performed throughout the analytic process. These issues may have influenced coding consistency and the interpretation of themes, particularly in a cross-cultural qualitative study.

Fifth, although thematic repetition was observed, saturation assessment remained partly subjective and may not have captured all relevant perspectives or variations within the study populations.

Finally, linguistic and cultural translation may have affected data interpretation. Interviews were conducted in Chinese or Japanese and analyzed in English. Although several strategies were used to reduce translation bias, including native-language transcription, collaborative coding, and participant checking, some shifts in meaning across languages and cultural contexts may have remained unavoidable.

Overall, these limitations should be considered when interpreting the findings. The results are intended to provide exploratory and context-specific insights rather than statistically generalizable conclusions.

## 5 Conclusion

Based on our findings, we propose a three-step collaborative co-governance framework to improve the medical environment and reduce disputes. By addressing root causes, it targets medical dispute antecedents to limit mediator interference (Figure 1) and curb downstream effects across three interdependent governance dimensions.

### 5.1 Alleviating financial burden

Reducing patients' financial burden is key to preventing cost-related disputes. Strengthening social protection for vulnerable groups prevents poverty-driven abandonment of treatment. Optimizing health insurance and resource allocation reduces institutional barriers, narrows regional disparities, and lowers care-seeking costs, easing doctors' workloads and improving DPRs. Limiting hospital commercialization, increasing public financing, reforming performance evaluation, and regulating excessive practices restore medical professionalism and rebuild trust.

### 5.2 Legal safeguards

A robust medical legal system and diversified dispute-resolution mechanisms are essential to curb violent disputes, ensure fair compensation, and standardize conduct. Stronger criminal laws should penalize patient-initiated violence, protect healthcare workers, and regulate media reporting to prevent antagonism. Laws must balance rights and responsibilities of doctors and patients while holding institutions accountable for misconduct. This creates a safer, transparent environment that encourages honest medical practice and fair dispute resolution.

### 5.3 Reshaping social morality and trust

Erosion of morality and trust in China reflects structural tensions from rapid socio-economic transformation, widening inequality, and institutional lag. Rebuilding trust requires sustainable education, cultivation of traditional values, and promotion of social ethics.

In conclusion, DPR problems and medical disputes are not isolated failures but embedded in broader social, medical, legal, economic, and cultural structures. Reducing disputes and improving DPRs in China demands systemic reforms: strengthening social security, refining healthcare and legal frameworks, and advancing ethics education. Rebuilding trust and fostering constructive moral practices is a long-term, systemic endeavor rather than a short-term task.

## Data Availability

The datasets presented in this article are not readily available because Our original data consists of 40 interview transcripts in Chinese or Japanese. Researcher may access to them at reasonable request. Requests to access the datasets should be directed to Xu Hua xuhua813hh@163.com.
